# Identification of transcriptional signals in *Encephalitozoon cuniculi *widespread among Microsporidia phylum: support for accurate structural genome annotation

**DOI:** 10.1186/1471-2164-10-607

**Published:** 2009-12-15

**Authors:** Eric Peyretaillade, Olivier Gonçalves, Sébastien Terrat, Eric Dugat-Bony, Patrick Wincker, Robert S Cornman, Jay D Evans, Frédéric Delbac, Pierre Peyret

**Affiliations:** 1 Clermont Université, Université d'Auvergne, Laboratoire: Microorganismes Génome et Environnement, BP 10448, F-63000 CLERMONT-FERRAND, France; 2 CNRS, UMR 6023, LMGE, F-63173 AUBIERE, France; 3 Clermont Université, Université Blaise Pascal, Laboratoire: Microorganismes Génome et Environnement, BP 10448, F-63000 CLERMONT-FERRAND, France; 4 CEA, DSV, IG, Génoscope, 2 rue Gaston Crémieux, 91000 Evry - France; 5 USDA-ARS, Bee research Lab, Beltsville - Maryland, USA

## Abstract

**Background:**

Microsporidia are obligate intracellular eukaryotic parasites with genomes ranging in size from 2.3 Mbp to more than 20 Mbp. The extremely small (2.9 Mbp) and highly compact (~1 gene/kb) genome of the human parasite *Encephalitozoon cuniculi *has been fully sequenced. The aim of this study was to characterize noncoding motifs that could be involved in regulation of gene expression in *E. cuniculi *and to show whether these motifs are conserved among the phylum Microsporidia.

**Results:**

To identify such signals, 5' and 3'RACE-PCR experiments were performed on different E. cuniculi mRNAs. This analysis confirmed that transcription overrun occurs in E. cuniculi and may result from stochastic recognition of the AAUAAA polyadenylation signal. Such experiments also showed highly reduced 5'UTR's (<7 nts). Most of the *E. cuniculi *genes presented a CCC-like motif immediately upstream from the coding start. To characterize other signals involved in differential transcriptional regulation, we then focused our attention on the gene family coding for ribosomal proteins. An AAATTT-like signal was identified upstream from the CCC-like motif. In rare cases the cytosine triplet was shown to be substituted by a GGG-like motif. Comparative genomic studies confirmed that these different signals are also located upstream from genes encoding ribosomal proteins in other microsporidian species including *Antonospora locustae*, *Enterocytozoon bieneusi*, *Anncaliia algerae *(syn. *Brachiola algerae*) and *Nosema ceranae*. Based on these results a systematic analysis of the ~2000 E. cuniculi coding DNA sequences was then performed and brings to highlight that 364 translation initiation codons (18.29% of total CDSs) had been badly predicted.

**Conclusion:**

We identified various signals involved in the maturation of E. cuniculi mRNAs. Presence of such signals, in phylogenetically distant microsporidian species, suggests that a common regulatory mechanism exists among the microsporidia. Furthermore, 5'UTRs being strongly reduced, these signals can be used to ensure the accurate prediction of translation initiation codons for microsporidian genes and to improve microsporidian genome annotation.

## Background

The genes of eukaryotes are generally considered to be monocistronic, each with its own promoter at the 5' end and a transcription terminator at the 3' end. The production of mRNAs from genomic DNA is so directed by sequences that determine transcript processing. Indeed, certain DNA motifs must be recognized during transcription to ensure transcriptional induction (promoter and regulator signals), splicing (donor and acceptor sites) and 3' end processing (polyadenylation and terminator signals) [1,2].

For transcription initiation, multiple cis-acting sequences are important for transcription. In eukaryotes, the core promoter serves as a platform for the assembly of a transcription pre-initiation complex (PIC) that includes transcription factors (TFIIA, TFIIB, TFIID, TFIIE, TFIIF, TFIIH) and RNA polymerase II, which function collectively to specify the transcription start site [3]. The core promoter encompasses the transcription start site and typically extends ~35 nt either upstream or downstream from the transcription start site. DNA elements that constitute this core promoter include the TATA box, the TFIIB recognition element (BRE), the initiator (Inr), and the downstream core promoter element (DPE). However, it is important to note that there is no universal core promoter element. Each of these motifs is found in only a subset of core promoters [4,5].

For splicing, practically all introns contain two highly conserved dinucleotides. The donor splice site has GT exactly after the point where the transcriptional machinery cuts the 5'-end of intron sequences and the acceptor site has AG exactly before the point where the intron 3'-end is cut [6]. Finally for mRNA 3' end processing, the polyadenylation signal plays a crucial role, since its mutation leads to a loss of mRNA through defective polyadenylation leading to unstable transcripts. Indeed, failure to terminate transcription through loss of poly(A) signals may also cause transcription read-through into downstream genes. The sequence of this signal, which is usually AAUAAA, is located ~20 nts upstream of the mRNA cleavage site [7].

Highly conserved signals also play a crucial role in translation, especially to define the initiation codon. Indeed, the start of translation in eukaryotes is governed by nucleotides flanking the AUG start codon which are essential for the right positioning of ribosomes. For example, the optimal context for initiation of translation in mammals is GCCRCCaugG [8].

Microsporidia were considered to be primitive amitochondriate eukaryotes. However, recent phylogenetic studies have consistently supported a placement of these organisms as a basal lineage of the fungi [9-12]. These parasites are characterized by small genomes ranging from only 2.3 Mb to 23 Mb [13,14], a trait reflected in the short length of most putative proteins compared to eukaryote orthologues and compact gene organization. Williams *et al*. [15] showed that transcripts encoding multiple genes are found in *Antonospora locustae*, a microsporidian species also characterized by a highly compacted genome (~5.4 Mbp) that has been partially sequenced [15]. By sequencing 1,146 cDNA clones, they showed that about 11% of cDNAs contained more than one gene. In addition, they found that a large number of transcripts harboured parts of flanking genes. This last finding suggested that control elements are displaced into or beyond adjacent genes, as a consequence of reduced intergenic regions. Using Northern blotting, RACE-PCR, and EST analysis approaches, Corradi *et al*. [16] recently reinforced that *A. locustae *cDNAs contain multiple genes. Furthermore they showed the presence of very large transcripts in sporal stages. Some of these transcripts were found to overlap up to four open reading frames in different strands. A comparative study performed by 5' and 3'RACE-PCR revealed that overlapping transcription was found in *E. cuniculi *in more than 80% of the analyzed loci and that this percentage is higher than that estimated for *A. locustae *[17]. These results could be justified because the genome of *E. cuniculi *is highly compact with a size of only 2.9 Mbp [18]. Concerning DNA signals implicated in transcriptional process, a cytosine triplet that is highly over-represented both in the honeybee pathogen *Nosema ceranae *and *E. cuniculi *has been highlighted within the 15 bp upstream of the start codon [19]. The authors also suggested that TATA-like promoters are important components of *N. ceranae *gene regulation.

In order to complement these previous studies and to finely characterize DNA signals governing the original transcription process in microsporidia, we first performed various RT-PCR coupled to both 5' and 3'RACE-PCR. These analyses were done using a locus from *E. cuniculi *consisting of six genes in close proximity located on both DNA strands. Based on these results we have then identified DNA signals that could play a role in 5' and 3' mRNA maturation. Furthermore, we extended the search of these specific signals on conserved genes encoding ribosomal proteins in the five fully or partially sequenced microsporidian genomes, *Encephalitozoon cuniculi*, *Antonospora locustae*, *Enterocytozoon bieneusi*, *Nosema ceranae *and *Anncaliia algerae *(syn. *Brachiola algerae*) [9]. These highly conserved signals were finally exploited to more accurately determine the translation initiation codon of all *E. cuniculi *CDSs. In this context, we propose that these conserved signals are a useful tool for efficient microsporidian CDS detection during genome annotation.

## Results and discussion

### Complete transcription of both DNA strands in *Encephalitozoon cuniculi*

**Figure 1:**
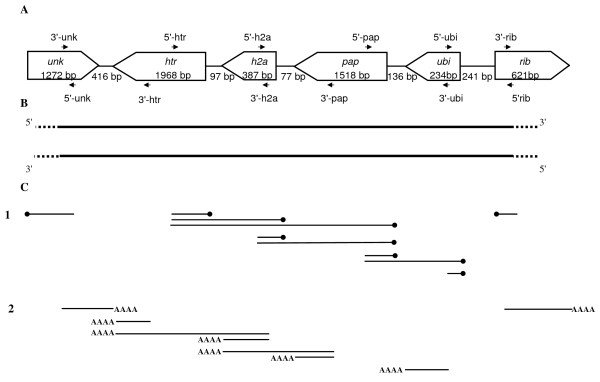
**Experimental evidences to explain transcriptional process in *E. cuniculi***. A: schematic representation of the genomic DNA region from the *E. cuniculi *chromosome 2 containing the genes *unk*, *htr*, *h2a*, *pap*, *ubi *and *rib*. White rectangles represent the position of the genes in genomic DNA (with gene names inside) and the arrowhead represents their transcriptional directions. The length in base pairs (bp) of the CDSs and the intergenic regions is also indicated. Black arrows represent the orientation of the specific primers used for RT, PCR and RACE-PCR experiments. B: results of RT-PCR experiments, indicating that both DNA strands are transcribed. Discontinuous lines indicate that respective transcription start site and transcription terminator one could not be defined. C: Schematic representation of RACE-PCR experiments. Black lines represent the length and position of the 5'-RACE (1) and the 3' RACE (2) fragments. PolyA tails are represented by AAAA and 5' end by a dot.

In order to better understand transcriptional regulation in microsporidia, we focused our attention on six *E. cuniculi *genes coding, respectively, a hypothetical protein (*unk*), the histone transcription regulator (*htr*), the histone H2A (*h2a*), the polyA polymerase (*pap*), the ubiquitin (*ubi*) and the 60 S ribosomal protein L7A (*rib*). Coding DNA Sequences (CDSs) of these genes are located on the chromosome II of *E. cuniculi *(ECU02_0700: CAD25100, ECU02_0710: CAD25101, ECU02_0720: CAD25102, ECU02_0730: CAD25103 and ECU02_0740i: CAD25104, ECU02_0750i: CAD25105). The two flanking genes of this DNA region (*unk *and *rib*) have the same orientation and the four other genes (*htr*, *h2a*, *pap *and *ubi*) are located on the other strand. These CDSs are separated by intergenic spacers of 416 bp, 97 pb, 77 bp, 136 bp and 241 bp, respectively (Figure 1A).

Using total RNA extracted from *E. cuniculi-*infected human cells, reverse transcription was performed using a primer specifically designed for the *rib *gene (5'-rib primer). PCR was then carried out using this primer and the 5'-ubi primer designed within the *ubi *CDS (Additional file 1). Sequence analysis of the amplified product demonstrated that rib transcript encompasses the ubiquitin gene region. To exclude a potential DNA contamination, PCR experiments were also carried out on RNAs without the reverse transcription reaction. No DNA amplification was observed in these controls (Additional file 1). Such mRNAs have previously been identified in *A. locustae *[15] and *E. cuniculi *[17] and the authors interpreted these results as run-on transcripts where termination signals are absent from the downstream intergenic regions and instead located within downstream genes. The authors also characterized cDNAs with long 5' ends corresponding to the antisense strand of CDSs indicating a movement of the transcription initiation faraway from the CDSs. In order to identify transcriptional regulators in *E. cuniculi *we then carried out fine mapping of the transcripts of the studied gene cluster.

PCR using 3'-htr and 3'-unk primers was then performed on reverse transcription products obtained with the 5'-rib primer located in the most distal gene of the cluster (Figure 1A). Sequence analysis of the PCR product indicated that the transcript encompasses at least the six studied genes (Figure 1B, Additional file 1). In this situation, the very large transcript harbours the sense sequence of the flanking genes *unk *and *rib *and the antisense sequence of the four other genes *ubi*, *pap*, *h2a *and *htr*. For the grasshopper microsporidian *A. locustae*, mRNA molecules harbouring several cistrons are also highly frequent with some transcripts corresponding to at least four genes [15]. A more recent study confirmed that large transcripts encompassing several CDSs are detected in *A. locustae *spores [16]. Our results confirmed that such transcripts are also present in developmental microsporidian stages which are likely transcriptionaly more active than spores. Indeed, in our experiments, total RNA extraction has been performed on the parasite during its developmental cycle in human cells.

In order to characterize mRNA including coding strands of *ubi*, *pap*, *h2a *and *htr *genes, a second reverse transcription experiment was carried out with the 3'-unk primer (Figure 1A). Using this RT product as template, a DNA fragment was then amplified with 5'-ubi and 5'-rib primers (Additional file 1). Sequence analysis confirmed the amplification of the intergenic region between *ubi *and *rib *genes thus demonstrating that the transcript may contain the sense sequences of the four internal genes and the antisense sequences of the two flanking genes (Figure 1B). All these results clearly indicated that both DNA strands corresponding to this *E. cuniculi *locus are completely transcribed. We then started the identification of the extremities of these large transcripts.

### Multiple forms of polycistronic and monocistronic transcripts coexist in *E. cuniculi*

To identify potential transcriptional start sites and the polyadenylation sites of the two large mRNA transcripts characterized above, RACE-PCR experiments were carried out (Figure 1C, Additional file 1).

**Figure 2:**
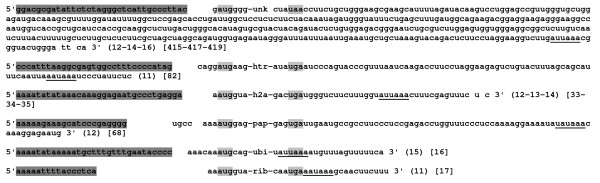
**Identification of 5' and 3' maturation sites for the six monocistronic genes**. 5' and 3'RACE-PCR products were generated using specific primers designed from the hypothetical protein (*unk*), the ubiquitin (*ubi*), the polyA polymerase (*pap*), the histone H2A (*h2a*) the histone transcription regulator (*htr*) and the 60S ribosomal protein L27A (*rib*). Sequences of 5' and 3'UTR are given. Translation initiation codon and stop codon are highlighted in light-grey for all genes. 3'UTR lengths are indicated in square brackets. Putative polyadenylation signals are underlined. Distances between putative polyadenylation signal and polyadenylation site are indicated in parenthesis. DNA sequences upstream transcription start sites that may represent core promoters are highlighted in dark grey. Complete CDS sequences are not given and are represent by gene names.

For the first mRNA harbouring the sense sequence of the flanking genes *unk *and *rib*, 5'RACE-PCR performed with the 5'-unk primer revealed only one DNA fragment (Figure 1C, Additional file 1). Sequence analysis demonstrated that this experiment amplify the 5' end of the *unk *gene and showed that the 5'UTR region is reduced to only 1 nt (Figure 2). Such result could not arise from a DNA contamination. Indeed in 5'RACE-PCR experiments, linker hybridization is dependent to reverse transcriptase step of mRNA. Double strand genomic DNA could not be used as matrix to ensure such hybridization.

In the same way, the mapping of the 3' end of the transcript using the 3'-rib primer in a 3'RACE-PCR experiment located the polyadenylation site 17 nts downstream of the *rib *stop codon. In this case, 3' RACE-PCR experiments also avoid DNA contaminations. Indeed reverse transcription is performed using oligo-d(T) primer to ensure only hybridization with all mRNA polyA tails. Furthermore, genomic DNA sequence analysis did not show potential adenine stretch that could be used to ensure oligo-d(T) primer hybridization during the PCR step.

Concerning the second transcript, corresponding to the other strand, 5'RACE-PCR using 5'-ubi primer also showed only one DNA fragment (Figure 1C, Additional file 1). Unexpectedly, analysis of the sequence allowed the characterization of the 5' end of the *ubi *gene positioned 6 nts upstream the putative AUG (Figure 2). Furthermore, 3'RACE-PCR using the 3'-htr primer permitted the identification of the polyadenylation site of the *htr *gene (82 nts downstream of the *htr *stop codon). These results are not in agreement with the identification of mRNA transcripts encompassing both the *unk *gene and *rib *genes based on our RT-PCR approaches. However, such transcript variability has been also described in *A. locustae*. Indeed, using Northern blotting, RACE-PCR and EST sequence analysis, Corradi *et al*. [16] identified different transcripts along a 6 kb region surrounding the actin locus.

In order to identify all potential transcripts covering the *E. cuniculi *DNA region from *unk *to *rib *genes, we then performed systematic 5' and 3'RACE-PCR experiments for the six genes (Figure 2). 5'RACE-PCR with the 5'-rib primer successfully characterized the 5' end of the *rib *gene and showed that the 5'UTR region is reduced to 2 nts. Moreover, sequence analysis revealed that the intron predicted during genome annotation is not spliced. It may be thus deduced that this is not a true intron or the intron is not spliced. In fact with a length of 27 nts, no frame shift is induced in the coding sequence of this ribosomal protein.

Using the 5'-pap primer, two RACE-PCR products were obtained (Figure 1C, Additional file 1). The sequence of the largest DNA fragment allowed us to confirm the 5' end of the *ubi *gene. But, unexpectedly, analysis of the smallest amplified sequence revealed the 5' end of the *pap *gene which is located 7 or 3 nts upstream of the putative AUG, depending on the clone product (Figure 2). With 5'-h2a and the 5'-htr specific primers, 2 and 3 RACE-PCR products were obtained, respectively. In each case, analysis of the smallest fragment allowed the characterization of 5' end of *h2a *and *htr *genes positioned at 1 and 4 nts upstream of their respective putative AUG (Figure 2). Sequencing of the higher fragments confirmed the 5' end of the *pap *gene for the two experiments and the 5' end of the *h2a *gene for the experiment performed with the 5'-htr primer. Similar results were obtained in *A. locustae *for the most abundant multigene transcripts encoding DNA polymerase α and ribosomal protein L24 [15]. Indeed, 5'RACE-PCR revealed two distinct transcripts: the first starting one base upstream of the ATG of the ribosomal protein L24 and the second one starting at the ATG of the DNA polymerase α gene. Corradi *et al*. [17] also observed this situation in *E. cuniculi *using two different primers located in genes coding respectively for a ring zinc finger transcription negative factor and for a 20S proteasome alpha-subunit. They identified the same 5' end in the upstream region of the second gene.

For 3' RACE PCR, using the 3'-h2a primer, two DNA fragments were amplified. Sequencing of the largest PCR products permitted to confirm the 3' end of the *htr *gene. Analysis of the smallest one revealed that the 3' polyadenylation site of the *h2a *gene was located 33, 34 or 35 nts downstream its stop codon (Figure 2). So, 3' RACE experiments confirmed that the histone *h2a *transcript is polyadenylated. Primary and secondary structure analyses performed on 3' UTR of this latter mRNA and on other putative members of the *E. cuniculi *histone family genes did not reveal a conserved sequence or a stem loop structure implicated in mRNA stability as is the case in metazoan organisms. Furthermore, genes coding for stem loop binding proteins [20] and U7 snRNA genes [21], required for the 3' end processing and the turnover of these mRNAs in metazoan species, were absent in the *E. cuniculi *genome. Therefore, final 3' end maturation of the histone family mRNA results only in a polyadenylation step to ensure their stability.

With 3'-pap primer, two RACE-PCR fragments were also obtained allowing the confirmation of the polyadenylation site of the *h2a *gene and the identification of the 3' end of the *pap *gene (68 nts downstream its stop codon). The polyadenylation site of the *ubi *gene (16 nts downstream from the stop codon) was also identified with the 3'-ubi primer. Finally, using the 3'-unk primer the polyadenylation site of the *unk *gene should be positioned 415, 417 or 419 nts downstream the stop codon (Figure 2).

Our results suggest that transcription in *E. cuniculi *is conducted on both stands leading to the formation of a mix of complex transcripts encompassing large regions with sense and antisense sequences corresponding to CDSs. For each transcript we noticed a precise mapping of their extremities in accordance to a faithful control of processing. We suppose that this high fidelity of processing should depend on conserved DNA regulatory signals.

### DNA signals implicated in 3' transcript processing

It should be noticed that Poly(A) signals on the transcript play a crucial role in 3' mRNA end formation in eukaryotic organisms. The sequence of this signal is usually AAUAAA and is located ~20 nts upstream of the mRNA cleavage site. However, signals are frequently degenerated and so may induce a bad recruitment of the cleavage/polyadenylation complexes [22].

3'RACE-PCR experiments were useful to characterize this putative polyadenylation signal for all the studied genes (Figure 2). In an attempt to generalize the presence of this signal in *E. cuniculi*, this sequence was systematically examined for all the genes located on chromosome II. However, in order to eliminate bias due to bad gene prediction obtained using *ab initio *approaches, we only focused our attention on genes coding for proteins with defined function (additional file 2). The consensus sequence AAUAAA has been identified near the stop codon for approximately half of the genes. Nevertheless, some other degenerated signal sequences which only differ from a single nucleotide have been also observed. For 25% of the observed cases, this degenerated sequence is AUUAAA. Sheets *et al*. [22], demonstrated that such signal reduces only weakly the polyadenylation and cleavage efficiencies compared to the AAUAAA sequence. However, the high frequency of such signals seems not to be a general rule in Microsporidia. In *A. locustae*, Corradi *et al*. [16] identified few 3'UTRs harbouring the consensus-like signal AAUAAA. They suggested that canonical polyadenylation sites can be identified but they are not common and apparently not frequently unused.

It should be noticed that for two genes, ECU02_0430 and ECU02_0530 (additional file 2), signals are more degenerate (AUUGAA and GAUGAA, respectively). In these cases, we cannot totally exclude that these two signals are functional. Polyadenylation signals could be more distantly related to the CDS. This is the case for *unk *gene with a 3'UTR spanning on 415, 417 or 419 nts (Figure 2). Also, we can not exclude the presence of functional signals in downstream CDS.

On the base of our experimental results (transcription of both strands leading to a mix of transcripts) and signal analysis, we propose a blurred 3' maturation of mRNA transcripts. This process implies a weak recognition of the polyadenylation DNA signal. In this situation, RNA polymerase should pass through the signal without stopping for the processing or recognize the signal for the cleavage and the polyadenylation steps leading to a mix of combinatorial transcripts as observed in our study.

To conclude, 5' and 3' RACE PCR experiments permit to identify mRNA on both strands that could encompass one or multiple CDSs. But in each case we could precisely and reproducibly identify 5' and 3' end processing sites of such molecules. Indeed, whatever the number of CDSs harboured by mRNAs, we have characterised the 5' end processing of the first CDS in close proximity of the translation start codon and the 3' end processing of the last CDS depending of the polyA signal.

Such conclusion could be also reinforced by the identification of large mRNAs fragments that encompass the 6 studied genes. Indeed, RT-PCR experiments have been performed to prove that both DNA strands are simultaneously transcribed leading to mono and polycistronic mRNA molecule resulting from a deficiency of accurate 3' end processing. In our example concerning reverse transcription carried out with 3'-unk primer, large mRNA molecule is the consequence of the absence of 3' end maturation of the *unk *gene. Maturation could arise using the next 3' end maturation site located in the *rib *gene or in a next downstream gene with the same orientation. So, theoretically, if we consider the first gene of each DNA chromosome strand, transcription may produce an mRNA molecule corresponding to the complete strand if all 3' end maturation sites are not recognized.

Moreover, in the *E. cuniculi *proteome, although the presence of all protein complexes involved in cleavage and polyadenylation was confirmed by *in silico *analysis, an interesting feature is the absence of the Pcf11 factor that could contribute to a low fidelity cleavage and polyadenylation processing steps [23]. The absence of this protein supports our model with a reduction in the fidelity or level of mRNA 3' end processing by reducing the signal recognition frequency.

In addition, these analyses appear to exclude the potential regulation of gene expression by the RNA interference process in *E. cuniculi*. This conclusion was also reinforced by the fact that genes coding for proteins implicated in such transcriptional regulation process have not been found in the *E. cuniculi *genome [16]. It should also be noticed that in eukaryotic organisms, gene expression regulation could involve natural antisense transcripts (NATs). These NATs are endogenous RNA molecules containing sequences that are complementary to other transcripts. NATs have been proposed to regulate the expression of their target genes by four mechanisms: transcriptional interference, RNA masking, double-stranded RNA (dsRNA)-dependent mechanisms and chromatin remodelling [24]. However, each mechanism requires different associations between sense and antisense expression patterns [24]. NATs have been detected in the transcriptomes of many protozoan parasites including *Plasmodium falciparum, Toxoplasma gondii*, *Theileria parvum*, *Trypanosoma brucei*, *Leishmania spp *and *Giardia lamblia *[25].

In *P. falciparum *and *T. gondii*, there is an intriguing negative correlation between the presence of sense and antisense RNAs indicating the potential for reciprocal negative regulation between NATs and coding transcripts. A prominent feature of transcription in *G. lamblia *is the abundant production of sterile antisense transcripts [26]. Such molecules are produced by promoters which ensure bidirectional transcription [27]. However, these authors suggested that NATs might simply result from unusual loose of transcription regulation in this organism. They are therefore not implicated in transcriptional regulations. In *E. cuniculi*, arguments such as that both strands should be transcribed and that a blurred maturation process should occur are in agreement with the exclusion of a putative control of gene expression by NATs.

### DNA signals implicated in 5' transcript processing

RACE-PCR experiments have shown a high reduction of 5'UTR regions (<7 nts). Such reduction may then suppose that promoter signals should be located in close proximity of the start of CDS. To confirm this hypothesis and identify these signals, we firstly analyzed the sequence upstream the transcription start for the 6 studied genes (Figure 2). These analyses confirm the presence of the CCC-like signal recently described by Cornman *et al*. [19]. However, this signal could not explain differential expression of genes because it seems present upstream of all the *E. cuniculi *genes [19]. In order to identify new regulation signals, we focused then our attention on the *rib *gene because it belongs to a multigenic family implicated in the same biological function. We suppose that all members of this family should be dependent on the same regulation process. After the analysis of the upstream ribosomal CDS sequence, a AAAAATTTT signal in close proximity of the cytosine triplet (1 nt) was identified and represents a putative regulatory motif for transcription. This motif was then searched on the complete chromosome II sequence and results showed that it is not randomly distributed. In fact, this signal is preferentially located in intergenic regions and more particularly upstream all the CDSs coding for ribosomal proteins (Table 1).

**Table 1:** Identification of conserved DNA signals shared by different microsporidian species potentially implicated in ribosomal protein gene expression.

Product name	*E. cuniculi*	*A. locustae*	*E. bieneusi*	*A. algerae*	*N. ceranae*
60S RIBOSOMAL PROTEIN L11	**AAAAAAAATTT**GAT**CCC**CAAC*	**AAAAATTT**ACCCT**CCC**	**AAAATTTTT**AT**GGT**TGTTTAA	**AAAATTTTT**AAC**CCC**ACA	**AAATATTTTTTTTTATTTTTT**A**CCC**TCTAAA

60S RIBOSOMAL PROTEIN L7A	**AAAAATTTT**A**CCC**TCAA	NS	**AAAAATTT**A**CCC**TAAAT	**AAAAAAATTT**A**CCC**	**AAATTTATTTTT**CTACC**CCC**AAAA

40S RIBOSOMAL PROTEIN S17	**AAAATTTTT**CAC**CCC**AAGA	**AAAAATTTTT**AG**CCC**TCT	**AAAATTTT**AAATCCTTACTTT	**AAAAATTTT**GTA**CCC**TCAT	**AACTATTTTT**A**CCC**TTCAAA

60S RIBOSOMAL PROTEIN L9	**AAAAAAATT**GTTAAATACCAGAAGGACTTAC**CCC**TT*	**AAAAAATTT**GAC**CCC**CG	**AAATTTTT**ACCCT	**AAAATTTTTT**AT**CCC**TT	**AATATTTTTTT**ATC**CCC**TAA

60S RIBOSOMAL PROTEIN L24	**AAATTTT**GGATGTGCTAA**GGG**TAAA	NS	NS	NS	**ATAATTTTTT**ATA**CCC**GTTAAA

40S RIBOSOMAL PROTEIN S8	**AAAAAAATTT**G**CCC**TGAA	**AAAAATTTTT**AC**CCC**	NS	**AAAATTTTTT**AAAAATGA**CCC**	**AATTTTTTTT**AC**CCC**ACAA

60S RIBOSOMAL PROTEIN L35A	**AAAATTTTT**GAT**CCC**TCGTT	NS	NS	NS	**AATTTTTTTT**ATC**CCC**TAAA

This table represents DNA sequences upstream the ATG codon for 7 ribosomal protein genes. isolated on the *E. cuniculi *chromosome 2 and their orthologs in *A. locustae, E. bieneusi, A. algerae*, and *N. ceranae*. DNA signals are highlighted as bold characters for AAATTT-like sequence and underlined characters for CCC- and GGG-like sequences. (NS indicates a Not already Sequenced gene and the star character stands for mispredicted start codon).

A complementary analysis of all the *E. cuniculi *genes coding for ribosomal protein was then performed, systematically allowing us to confirm the presence of a AAATTT-like signal in the upstream intergenic space (additional file 3). Such a palindromic sequence could also play a transcriptional role for two divergent transcriptional units. This could be the case for ribosomal protein S16 (ECU03_310) and L13 (ECU03_320) CDSs, which present a head to head gene organization and are only separated by a 35 bp intergenic space containing this AAATTT signal.

Our analysis demonstrated that for 3 genes coding respectively for 60S L24, L10A and L4 the CCC-like motif located between the AAATTT signal and the translational start codon could not be identified (Table 1 and additional file 3). However, an alternative GGG-like motif that could probably play the same function is present. Such signal was also identified by Cornman *et al*. [19] and used to perform their promoter analyses in *Nosema Ceranae*, a microsporidian pathogen of honeybees. Indeed, for NcORF-01850 and NcORF-00057 genes of *N. ceranae *and gi|19173514, gi|19173508 and gi|19074866 genes of *E. cuniculi*, the CCC motif is absent but we could characterize the GGG motif in the 15 bp upstream the start codon [19].

In order to determine whether this signal is widespread among microsporidian species, a comparative genomic study was performed between *E. cuniculi *[15], *A. locustae *[28], *E. bieneusi*[29], *N. ceranae *[19] and *A. algerae *(personal data) for genes coding for ribosomal proteins (Table 1 and additional file 3). Systematically, for all genes the AAATTT-like signal was identified. Concerning the CCC motif, it was identified between the AAATTT sequence and the translational transcription start codon for all *A. locustae *and *A. algerae *genes. For *E. bieneusi *and *N. ceranae *respectively one and two genes harbour the GGG-like alternative motif instead of the CCC one (Table 1 and additional file 3). Such signal for *N. ceranae *has been also found for the same genes in *E. cuniculi*.

These results suggested that transcriptional regulation of this multigenic family is under control of conserved regulatory elements in microsporidia. Our genomic analysis included various species which belong respectively to clade II (*A. locustae*), clade III (*E. cuniculi*, *E. bieneusi *and *N. ceranae*) and clade V (*A. algerae*) of the Microsporidia [30]. In addition, these species are respectively characterized by genomes with highly variable AT percent, (47% for *A. locustae*, 47% for *E. cuniculi*, 74% for *N. ceranae*, 75% for *E. bieneusi *and 76% for *A. algerae *(personal data). However, such variability does not seem to influence the DNA motif implicated in transcriptional process.

Ribosomal proteins are over-synthesized in cells to ensure translation steps. We can suggest that the signal regulating ribosomal proteins should also be located upstream from highly expressed genes. Using a proteomic approach, Brosson *et al*, [31] have identified 32 highly abundant proteins in *E. cuniculi*. For six genes (ECU02_0100, ECU04_1100, ECU05_0510, ECU05_0590, ECU09_1250 and ECU09_1820) coding for these proteins we could then characterize an AAATTT-like motif upstream from the AUG codon (additional file 4). Nevertheless, for the other ones, we identified an adenine-rich sequence in most cases. Such sequence was also present upstream the *ubi*, *pap *and *h2a *genes coding for proteins that likely need to be highly expressed in eukaryotic cells. For *htr *and *unk *genes such bias could not be detected. However, for those last genes, upstream CDS regions present higher cytosine and guanine percentages, and we could locate multiple CCC or GGG motifs. Such analysis is in agreement with alternative commonly found eukaryotic promoters. The architecture of promoters uses a distinct combination of core promoter elements and plays a critical role in regulating gene expression. Furthermore, both the AAATTT and adenine-rich region may play a similar role as TATA box and it was shown that this signal not only specifies the initiation for transcription, but also acts as regulatory element for enhancer function [32]. In our case the different TATA-like sequences may then be involved in differential gene regulation.

Our results have shown that transcriptional regulation signals are located in close proximity to 5' CDS ends. However, Corradi *et al*. [17] have also studied by 5'RACE-PCR different genes in *E. cuniculi*. They could then identify 5'-RACE products that may begin at different positions highly distant from the start codon. Such results were described for ECU4_1410, ECU10_1600 and ECU10_1740. Nevertheless, analysis of the neighbouring upstream regions allowed the identification of the cytosine triplet visible after adenine rich sequences (additional file 5) that represent unambiguous promoter sequences. To explain their results we could suggest the presence of cryptic promoter sites that could then be used to initiate the transcription process. This conclusion was validated by 5'RACE-PCR experiments on the *pap *gene where different CCC- or GGG-like motifs are in agreement with the characterization of two transcriptional start sites.

In summary, transcriptional gene regulations in Microsporidia seem to be dependent to the presence of conserved signal sequences upstream the CDSs.

### Re-annotation of the *Encephalitozoon cuniculi *genome

To validate the importance of these signals in the transcriptional process of all *E. cuniculi *genes, a systematic analysis of regions located upstream of all the CDSs identified in the genome was then performed (additional file 5). It was based notably on the identification of CCC- or GGG-like motif. Such signals have been identified near the AUG codon for 1591 genes (1474 with the CCC motif and 117 with the GGG one).

The genome of *E. cuniculi *consisting of 1996 CDSs, two hypotheses could explain the absence of this signal for the 405 other genes: (1) these genes are regulated by different signals which are not conserved, (2) prediction of translational initiation start sites are erroneous. Gene predictions have been performed automatically during *E. cuniculi *genome annotation and the choice of AUG codon was systematically made in favour of the first one encountered into the open reading frame.

The characterization of the conserved signals for genes coding for ribosomal proteins allowed us to highlight mispredicted initiation start codons suggesting some problems for a reliable annotation of some CDSs. Indeed, for the 72 genes coding ribosomal proteins, 13 showed a badly predicted start codon (additional table 1). Moreover, these analyses allowed to identify 3 supplementary introns. It should be taken into account that such correction has been suggested for gene coding the 60S ribosomal protein L6 [19]. In order to validate hypothesis of bad predicted start codons, other potential AUG codons were characterized and their upstream sequences analyzed. This approach allowed us to bring to light the presence of AUG with CCC-like signal for 346 genes and the GGG one for 18 genes (additional file 5). In order to validate the choice of these new predicted translation initiation codons, two complementary approaches were performed. The first one was based on a comparative analysis of the N-terminal region of proteins coding by these genes using a BLASTP approach [33]. Protein alignments with orthologous sequences of the NCBI nr database were helpful to validate corrections for 187 genes. For the other ones, absence of orthologous proteins in databases or the presence of highly variable N-terminal did not allow to validate the new predictions. A second approach, resting on Kozak sequence analysis, was then implemented. The sequence located around the AUG codon presents a high degree of conservation for all genes of the same organism. We first identified this sequence using the 1474 correctly predicted CDSs with CCC-like motif in upstream regions (Table 2). No bias could then be detected upstream from the AUG codon which is in agreement with a highly reduced 5'UTRs in *E. cuniculi*.. Nevertheless, we have identified a strong bias for adenine (37.65%) and guanine (40.57%) in the +4 position and a significant one for adenine (45.32%) in the +5 position (Table 2). For the AUG considered as badly predicted, the same study did not show such biases (Table 2). When we consider newly predicted AUG, Kozak sequence analysis shows the same biases in favour of adenine and guanine in position +4 and adenine in position +5 respectively for initiation start codon validated or not validated by BLASTP. Finally, we performed independent analysis of AUG (right predicted or corrected) with GGG-like motif in upstream regions and same biases could then be identified (Table 2). It should be noticed that for 41 genes we could not locate the CCC- or the GGG-like motif. Furthermore, for these genes, no putative polyadenylation signal could be characterized. We could then suppose that these genes coding for proteins with unknown function and with no ortholog in other organisms correspond to erroneously predicted genes. Using our corrections, we also showed that overlapping CDSs in head to head or head to tail organizations are very rare in *E. cuniculi*. Indeed, base on first annotation [18], 129 overlapping of CDSs have been defined in such organizations. Nevertheless, correction of start codons allowed us to eliminate 120 of them. Such result is also in agreement with the presence of transcriptional signal in intergenic regions in most cases. For the 9 others, the unambiguous identification of transcriptional signals showed that they may be present in rarely cases in adjacent CDSs.

**Table 2:** Study of Kozak sequences for correctly and mispredicted start codons.

POSITION	NUCLEOTIDE	correctly predicted AUG with CCC-like motif (1474)	mispredicted AUG (364)	corrected AUG and BLASTP validated (187)	corrected AUG but not BLASTP validated (177)	correctly predicted (117) and corrected (18) AUG with GGG-like motif
A						
						
T						
						
G						

+4	A	**37.65**	**21.15**	**41.71**	**36.16**	**48.15**
	
	T	10.92	22.53	10.16	11.86	7.41
	
	G	**40.57**	**30.49**	**41.714**	**42.94**	**34.81**
	
	C	10.85	25.82	6.42	9.04	9.63

+5	A	**45.32**	**25.27**	**47.06**	**45.76**	**37.04**
	
	T	19.54	28.57	14.44	15.82	27.41
	
	G	19.74	18.68	20.32	19.77	22.96
	
	C	15.4	27.47	18.18	18.64	12.59

+6	A	26.87	24.73	28.88	27.12	32.59
	
	T	23.47	32.14	20.86	23.16	23.7
	
	G	28.7	22.53	26.2	30.51	28.15
	
	C	20.96	20.6	24.06	19.21	15.56

Nucleotide frequencies of occurrence downstream (+4 to +6) the ATG codons Significant nucleotide biases are indicated in bold

Our analyses also highlighted the importance of these signals to carry out CDS prediction during microsporidian genome annotation. For genes coding ribosomal proteins in *A. locustae *and *E. bieneusi*, we were able to detect respectively 5 and 7 mispredicted translational initial codons (additional file 3). For *A. locustae *this study allowed the identification of one intron in the 40S ribosomal S6 gene that is also present in the *E. cuniculi *ortholog.

## Conclusion

This study provides new evidences for transcriptional regulation of microsporidian genes. RT-PCR and RACE-PCR approaches were able to demonstrate that both DNA strands at a same location should be transcribed for *E. cuniculi *species. To explain these results, attention was particularly focused on DNA signals implicated in this transcription process. Relying on these analyses, we propose a new transcriptional regulation process which implies the blurred recognition of the AAUAAA-like signal involved in 3' end maturation of mRNAs. When this signal is not recognized, the transcription process continues and leads to the formation of mRNAs including multiple CDSs in various orientations. In the contrary case, identification of this signal leads to 3' end maturation.

Concerning the transcriptional initiation process we suggest a control linked to the presence of multiple conserved DNA signals located in close proximity of the start codon. Systematic analysis of the whole *E. cuniculi *genes and genes coding for ribosomal proteins in *A. locustae*, *E. bieneusi*, *N. ceranae*, and *A. algerae *allowed us to characterize a CCC-like or an alternative GGG-like signal that is rarely found to be degenerated on one base. These signals are generally found after adenine- and/or thymine-rich regions. For the five studied species, an AAATTT-like motif is always found for genes coding for ribosomal proteins. This signal was also found upstream genes coding for highly expressed proteins in *E. cuniculi*. These signals have been then used to ensure re-annotation of *E. cuniculi *CDSs. Using this approach, we were able to correct more than 18% of the previously predicted CDSs, confirming that these signals represent tremendous tools for accurate microsporidian genome annotation.

## Methods

### Cell culture

1 10^9 ^spores of *E. cuniculi *harvested on HFF cells culture were used to infect during 2 hours flask (75 cm2) containing HFF host cells grown at confluence. Cultures were then washed three times with PBS (1X) to eliminate spores that did not invade host cells and incubated during 2 days as previously described [34].

### RNA extraction

Total RNA was extracted after 2 days *E. cuniculi-*infected HFF using RNeasy Midi Kit (Qiagen). Before extraction, infected cells were washed three times with 5 mL of PBS to eliminate extracellular spores. RNA quality and quantity were estimated using the Agilent bioanalyser with the RNA 6000 Nano LabChip^® ^Kit. To remove DNA contamination 5 μg of previously extracted RNA were incubated with 20 U of Dnase I (Boerhinger, Mannheim) at 30°C for 30 min in a final volume of 10 μl. Enzymatic activity of DNase I was then inhibited by addition of 1/10 volume of 0.2 M EDTA.

### Primer design

Primer design was performed using a modified program based on the algorithm developed by Rimour *et al*. [35]. Two primers were then defined for each of the six targeted genes (Table 3) and were used for RT-PCR and RACE-PCR experiments. These primers are highly specific of the targeted genes, avoiding potential cross hybridization with other *E. cuniculi *transcripts or host ones.

**Table 3:** List of oligonucleotide primers used for RT, PCR and RACE-PCR experiments.

GENE	ORF NUMBER	PRIMER NAME	SEQUENCE (5'-3')	POSITION ON CHROMOSOME SEQUENCE
*unk*	ECU02_0700	5'-unk	ATGCCATCTCGGCACTTAGAAGGCTC	complement (86499..86524)
		3'-unk	GCGCTCCAGCTCATCAGCGGCGAG *#	86443..86466

*htr*	ECU02_0710	5'-htr	ACCTGTCCGTCCATACCACGTTGC	88604..88627
		3'-htr	GATACCGACCTGGCGCTTGAACACG #	complement (87230..87254)

*h2a*	ECU02_0720	5'-h2a	TGATCTCGCTGATTAGGTACATTAC	89217..89241
		3'-h2a	GGACCAGGATGAGGATATCGAAGG	complement (89268..89291)

*pap*	ECU02_0730	5'-pap	TCAAAGAACCCACGCTCCTGTAGG	90874..90897
		3'-pap	GTCAAAGTTCGAGGCGGTTGACGAC	complement (89812..89836)

*ubi*	ECU02_0740i	5'ubi	CTCGATACTGTCCGAAGGCTCGACT #	91300..91324
		3'-ubi	AGTCGAGCCTTCGGACAGTATCGAG	complement (91300..91324)

*rib*	ECU02_0750i	5'-rib	TGTCATGCTCTGACAGAACCGTCC *#	complement (91758..91781)
		3'-rib	AGAAGAAGGTATCCAAGCTGGCC	91698..91720

For each gene two specific primers were designed. Each of them was used for RACE-PCR. * and # characters indicate the primers used for RT and PCR, respectively. ORF number are also indicated.

### RT-PCR experiments

The reverse transcription reactions were carried out at 42°C for 2 h with 5 μg of RNA extracted from *E. cuniculi*-infected cells in a final volume of 20 μl with 100 U of SuperScriptIII reverse transcriptase (Invitrogen), 1 U of RNasin+ Inhibitor (Promega), 0.25 mM of dNTPs (Invitrogen), 0.1 M DTT (Invitrogen) and 0.625 μM of primer (Table 1). Two different reverse transcription experiments were performed independently with one specific primer defined in ECU02_0750i (60S ribosomal protein L7A; CAD25105) and in ECU02_0700 (hypothetical protein; CAD25100) genes respectively (Table 1). The first primer sequence corresponds to the coding strand and the second one to the non coding one. The reverse transcription (RT) products were then subjected to PCR amplifications using Advantage 2 PCR kit (CLONTECH) following the manufacturer's instructions. The final PCR reaction mixture of 25 μL consisted of 1 μL of cDNA, 1 μM dNTPs, 1 μM of corresponding primers and 2 units of Taq polymerase. Five minutes at 94°C were followed by 30 cycles of 94°C for 15 s, annealing temperature (68°C) for 15 s and 72°C for 90 s. After the final cycle, the temperature was maintained at 72°C for 7 minutes to allow completion of synthesis of amplified products. The PCR products were then visualized on ethidium bromide stained-agarose gels (1%). DNA fragments were purified with the QIAquick Gel Extraction kit (QIAGEN) and cloned into the pGEM^®^-T Easy Vector (PROMEGA). One clone for each PCR product was then sequenced with the Big-Dye^® ^Terminator v1.1 kit (APPLIED BIOSYTEMS) on the ABI PRISM 310 Genetic Analyzer (APPLIED BIOSYTEMS) according to recommendations of these different manufacturers.

To exclude amplification of contaminating genomic DNA, control reactions were performed by direct amplification of total RNA.

### RACE-PCR experiments

5' and 3'RACE analyses were carried out to amplify cDNA ends of putative operons using the SMART™ RACE Amplification kit according manufacturer recommendations (CLONTECH). Reverse transcription steps were performed with 300 ng of *E. cuniculi *total RNA extracted from infected cells using the modified oligo-d(T) primer provided by the SMART™ RACE Amplification kit. This RT product was then used for PCR amplifications with specific gene primers (Table 1). PCR products corresponding to all amplified DNA bands were then extracted, purified, cloned and sequenced as described above. Five clones for each PCR product were sequenced.

### Bio-analysis strategy for DNA signal identification

DNA signals implicated in 5' and 3' mRNA maturation were manually identified in *E. cuniculi *genome using Artemis software [36]. EMBL files have been then updated after translational initiation codon correction.

A BLASTP approach was implemented to identify orthologous ribosomal proteins from *E. cuniculi*, *A. locustae*, *E. bieneusi *and *A. algerae *and then performed a comparative genomic analysis of DNA motifs. DNA sequences were respectively retrieved from EMBL database (*E. cuniculi *and *E. bieneusi*) and from *A. locustae *Genome Database [28]. For *A. algerae *(personnal data), sequences were extracted from data generated by the *A. algerae *whole genome sequencing project which was initiated in collaboration between Genoscope (Evry, France) and the laboratory "Microorganismes: Génome et Environnement" (UMR CNRS 6023, France) [14].

## Abbreviations

RACE: rapid amplification of cDNA end; UTR: untranslated region; CDS: coding DNA sequence; NAT: Natural Antisense Transcript; PIC: Transcription Pre-initiation Complex.

## Authors' contributions

EP, OG, PW, PP carried out the experimentations and have participated to analysis and interpretation of data. EP, EDB, ST and OG performed *in silico *analysis. EP, OG and PP planed the study and wrote the manuscript. RSC, JDE, FD and PP have given final approval of the version to be published. All authors read and approved the final manuscript.

## Supplementary Material

Additional file 1 **Experimental evidences to justify transcriptional process in *E. cuniculi***. A: results of RT-PCR experiments after electrophoresis migrations. RT experiments were performed respectively using 5'-rib primer (line 1 and 5) and 3'-unk primer (line 3 and 7). PCR experiment were then realized using 5'-ubi and 5'-rib primers (line 1, 2, 3 and 4) and 3'-htr and 3'-unk primers (line 5, 6, 7 and 8). PCR experiments were carried out on RNAs without the reverse transcription reaction (line 2, 4, 6, 8) to exclude a potential DNA contamination. Some Positions of *HindIII*/*EcoRI *fragments (lines E) are shown at left in bases pairs. B: Results of 5' and 3' RACE-PCR experiments performed respectively on *ubi*, *pap *(B1), *h2a*, *htr *(B2), *rib *and *unk *(B3) genes. Specific RACE fragments were put into white square frames. PCR aspecific fragments were also cloned and sequenced. Results showed that did not correspond to targeted genes. Such non specific amplifications are frequently observed in RACE-PCR experiments because only one specific primer is used for PCR reaction and the other one may hybridize with all mRNA molecules. Furthermore in the sample, mRNA from *E. cuniculi *but also from HFF host cells are both presents enhancing aspecific amplification. For B3 gel, well 2 and well 4 correspond to controls: 5' RACE-PCR performed with 5'-*rib *on 3' DNA matrix and 3' RACE-PCR perfomed with 3'-*rib *on 5' DNA matrix. Some Positions of ladders are shown at left in bases pairs. Click here for file

Additional file 2 **Identification of putative polyA signals for *E. cuniculi *genes located on chromosome II and coding for proteins with defined function**. First column indicates protein systematic name, second column presents ORF number, third column shows the putative polyA signal sequence, and the last column indicates the distance to the stop codon. When negative distances are used it indicates that the AAUAA-like motifs begin in their respective CDSs. Click here for file

Additional file 3 **Characterization of DNA signals implicated in ribosomal protein gene expression shared by microsporidian species**. First column indicates product name, the five other columns present DNA sequences upstream the AUG start codon for *E. cuniculi*, *A. locustae*, *E. bieneusi*, *A. algerae *and *N. ceranae*. AAATTT-like motifs are colored in blue, CCC-like motifs in red and GGG-like motifs in green. The star character stands for a mispredicted start codon, and NS stands for not already sequenced genes. When a gene contains an intron it is indicated within brackets. Click here for file

Additional file 4 **Upstream signals identification of genes coding for highly abundant proteins in *E. cuniculi *spores **[28]. First column indicates ORF number, second column presents protein function, and third column the DNA upstream the AUG start codon. Adenine-rich regions are indicated in blue, CCC-like motifs in red and GGG-like motifs in green. The star character stands for a mispredicted start codon. For the following genes, ECU05_1590, ECU08_1610 and ECU11_0530, adenine-rich region was not present. Nevertheless, we can observe CCC- and/or GGG-like multiple motifs in these regions. Click here for file

Additional file 5 **Identification of upstream signals for total *E. cuniculi *genes after start codon correction**. First column indicates ORF number. Second column presents signal sequence upstream the AUG start codon where CCC-like motifs are colored in red, GGG-like motifs in green, and MP stands for mispredicted genes (not a real gene). The third column indicates the new position of the AUG start codon when it has been corrected. BV indicates that corrections have been validated by Blast approach. Click here for file
