# Supplementary figures and images for: Identification of transcriptional signals in Encephalitozoon cuniculi widespread among Microsporidia phylum: support for accurate structural genome annotation
Source: BMC Genomics. 2009 Dec 15;10:607. doi: 10.1186/1471-2164-10-607 (PMC2803860; doi:10.1186/1471-2164-10-607)

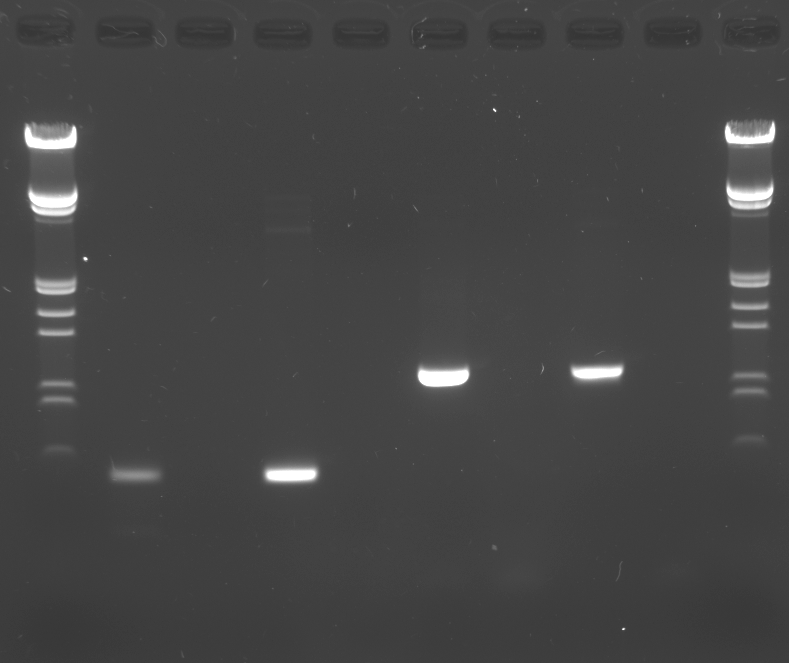


E 1 2 3 4 5 6 7 8 E

**A**

E 1 2 3 4 5 6 7 8 E

1584

947

564


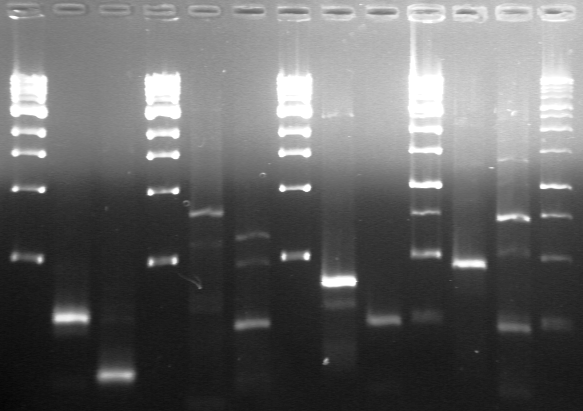

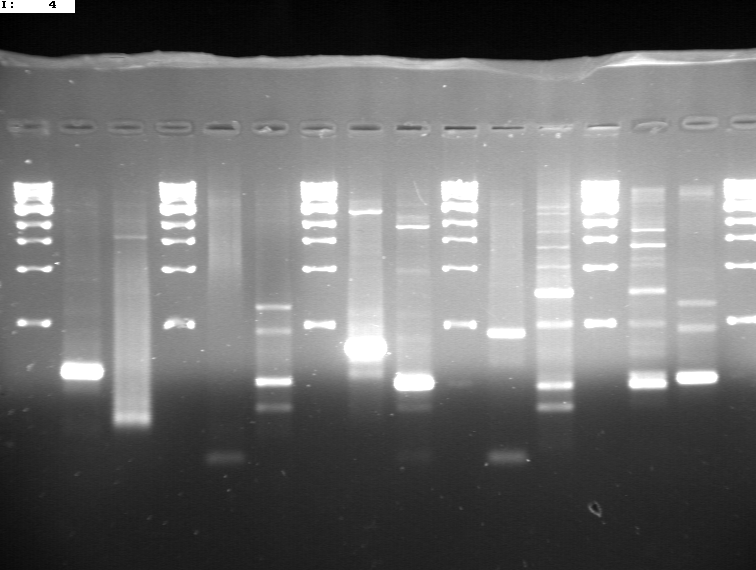


5’

3’

*ubi*

*pap*

3’

5’

**1**

**2**

**B**

*h2a*

*htr*

3’

3’

5’

5’

2000

3000

3000

2000

1000

1500

1000

1500

500

500

**3**


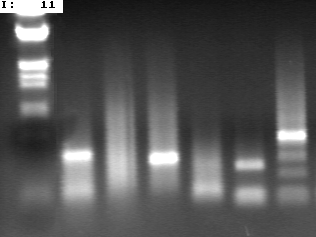


*rib*

*unk*

3’

3’

5’

5’

947

1584

564

125

Supplement: Additional file 5 — Identification of upstream signals for total E. cuniculi genes after start codon correction. First column indicates ORF number. Second column presents signal sequence upstream the AUG start codon where CCC-like motifs are colored in red, GGG-like motifs in green, and MP stands for mispredicted genes (not a real gene). The third column indicates the new position of the AUG start codon when it has been corrected. BV indicates that corrections have been validated by Blast approach. [file 1471-2164-10-607-S5.DOC]
